# Cholesterol Accumulation Caused Oxidative Stress Associated With Impaired Antioxidant Capability and Mitochondrial Function in the Liver of Nile Tilapia

**DOI:** 10.1155/anu/7643962

**Published:** 2025-08-30

**Authors:** Zhixiao Liu, Yi Xiao, Jiaying Xie, Huiwen Zhang, Qiming Huang, Óscar Monroig, Douglas R. Tocher, Xiaojuan Liu, Fan Lin, Cuiying Chen, Shuqi Wang, Ruixin Li

**Affiliations:** ^1^Guangdong Provincial Key Laboratories of Marine Biotechnology, Shantou University, Shantou 515063, China; ^2^Institute of Aquaculture Torre de la Sal (IATS), CSIC, Ribera de Cabanes, Castellón 12595, Spain

**Keywords:** cholesterol accumulation, liver, mitochondria, Nile tilapia, oxidative stress

## Abstract

In mammals, cholesterol accumulation in tissues often results in health damage, such as oxidative stress. In contrast, the adverse effects of cholesterol accumulation on the physiological health of fish remain largely unexplored. The present study investigated the impacts of cholesterol accumulation on oxidative stress and the potential mechanisms involved in Nile tilapia (*Oreochromis niloticus*). Thus, Nile tilapia were fed either a control diet (C) or a high-cholesterol (1.6%, HC) diet for 8 weeks. The viscero-somatic (VSIs) and hepatosomatic indices (HSIs) were increased significantly in fish fed the HC diet and, in accordance, HC intake caused the accumulation of cholesterol in the liver, intestine, head kidney, and spleen. Intake of the HC diet, affected oxidative stress as evidenced by elevated malondialdehyde (MDA) levels in the liver and head kidney and reduced catalase (CAT) activities in the liver and spleen, while SOD activities were increased in the spleen and intestine. Moreover, cholesterol accumulation induced endoplasmic reticulum (ER) stress, inflammation, and apoptosis in the liver and head kidney, as evidenced by increased expression levels of key genes. Metabolome analysis indicated that metabolite levels in tilapia fed the HC diet were primarily enriched in glutathione metabolism and the tricarboxylic acid cycle (TCA), with significantly reduced levels of glutamine, glutamate, glycine, citrate, isocitrate, aconitate, malate, and oxalate. In addition, transmission electron microscopy (TEM) analysis showed accumulation of lipid droplets and distinct alterations in the morphology of mitochondria within hepatocytes of tilapia fed HC. Moreover, significantly increased serum alanine aminotransferase (ALT) and aspartate aminotransferase (AST) activities were found in fish fed the HC diet. Overall, the data suggested that HC intake induced oxidative stress, which might be associated with impaired antioxidant capability and mitochondrial function, as mitochondria are the primary site of producing cellular reactive oxygen species (ROS). The present study is the first to report the impacts of cholesterol accumulation on oxidative stress and health damage in fish, and suggested targeted cholesterol-lowering interventions as a promising therapeutic strategy for addressing health issues in aquatic animals.

## 1. Introduction

Currently, aquaculture faces a notable obstacle due to the scarcity and increasing prices of fishmeal, which impede its continuing development. Thus, high-energy/fat diets and/or high-carbohydrate diets are commonly utilized for their protein-sparing effect to enhance fish growth. However, these diets often cause adverse effects, such as excessive accumulation of triacylglycerol and cholesterol, and health damage in fish [1–3]. Previous studies concentrated primarily on the adverse effects caused by triglyceride accumulation but generally ignored the health consequences of cholesterol accumulation [1–3]. Despite cholesterol being essential in various physiological processes and is distributed widely throughout the body, cholesterol accumulation often increases the risk of various diseases, such as cardiovascular and metabolic syndrome and immune system issues in mammals [4, 5]. Therefore, excessive cholesterol frequently exacerbates disease progression and is a significant “marker,” since it can trigger oxidative stress, inflammation, and cell apoptosis via diverse signaling pathways. In fish, it was reported that supplementation of 1.5% cholesterol in soybean meal-based diets showed growth-promoting effects in channel catfish (*Ictalurus punctatus*) and turbot (*Scophthalmus maximus*) [6, 7], although elevated cholesterol levels in serum and liver in channel catfish also raised concerns about potential health implications [7]. Further research suggested that dietary cholesterol supplementation might exacerbate arteriosclerotic lesions and the incidence of fatty liver in fish [8], which emphasized the need for cautious consideration of the adverse effects of cholesterol accumulation on fish health. Indeed, we revealed recently that cholesterol accumulation caused metabolic disorders and health damage in tilapia (*Oreochromis niloticus*), although the precise metabolic mechanisms of how cholesterol accumulation impaired fish health remained unclear [9].

Cholesterol is crucial for modulating the structural and functional properties of membrane bilayers, influencing various cellular organelles, including mitochondria. Despite mitochondrial membranes having relatively low cholesterol content compared to other cellular membranes, excessive accumulation can disrupt mitochondrial function through several mechanisms. These disruptions include limiting critical antioxidant systems, including glutathione homeostasis, increasing reactive oxygen species (ROS) production, and impairing the assembly of respiratory supercomplexes [10–12]. For example, high-cholesterol (HC) treatment impaired mitochondrial structure and function, leading to intracellular oxidative damage in human regulatory T cells [13]. Additionally, cellular oxidative stress can trigger endoplasmic reticulum (ER) dysfunction, resulting in misfolded protein accumulation and elevated ER stress [14]. When oxidative stress exceeds the threshold of defense capability, cells transition to apoptotic pathways to initiate cell death [15, 16]. It should be noted that in fish, high-carbohydrate or high-fat diets often result in inflammation, oxidative damage, and apoptosis. For example, a high-starch diet led to hepatic lipid deposition, oxidative stress, and inflammation, while a high-fat diet caused oxidative stress, lipid peroxidation, and liver impairment in Nile tilapia [17]. However, there is a conspicuous gap in knowledge of the adverse physiological effects associated with cholesterol accumulation, which is frequently found in fish fed high-energy diets [1–3]. This limitation has constrained the development of strategies for lowering cholesterol, which is detrimental to the growth and health of aquatic animals.

Nile tilapia, a globally cultivated and economically important fish species, has emerged as an ideal model for aquatic animal nutrition metabolism research [9, 18]. Based on our preliminary findings, cholesterol accumulation had been identified as a causative factor in metabolic dysregulation and impairment of health in tilapia [9]. The aims of the present study were to assess the impact of cholesterol accumulation on oxidative stress and the potential protective mechanisms that exist in fish. Nile tilapia were fed a normal low-cholesterol control diet (C) or a 1.6%, HC for 8 weeks, and cholesterol content, lipid peroxidation, and antioxidant capacity in various tissues, as well as ER stress, inflammation, apoptosis, and liver health of fish, were evaluated. In addition, transcriptome and metabolome analyses were conducted to elucidate the biochemical and molecular mechanisms underlying the health damage induced by cholesterol accumulation in fish, with a particular focus on antioxidant capability and mitochondrial function in the liver. The study was the first to reveal mechanisms underpinning adverse effects of cholesterol accumulation on fish physiological health and suggested targeted cholesterol-lowering interventions as a promising therapeutic strategy for addressing health issues in aquatic animals.

## 2. Materials and Methods

### 2.1. Experimental Diets

Our previous study showed that 1.6% dietary cholesterol inclusion represented a HC diet and was suitable for constructing the model of cholesterol accumulation in Nile tilapia [9]. Thus, two experimental diets, a C (unsupplemented) and a HC diet (supplemented with 1.6% cholesterol), were formulated with casein as the primary protein source, while corn starch and soybean oil served as carbohydrate and lipid sources, respectively (Table 1). The diets were designed to maintain protein and carbohydrate levels at approximately 39% and 32%, respectively, consistent with our previous research [9]. Cholesterol with a purity exceeding 99.0% (Glpbio, CA, USA), was incorporated into the diet HC formulation at the expense of cellulose. Briefly, the dry raw ingredients were ground and passed through a 40-mesh sieve, accurately weighed according to the diet formulation and mixed in a blender. Initially, the micronutrients, including minerals, vitamins, and sodium carboxymethyl cellulose, were premixed, and then the macronutrients, such as fishmeal, soybean meal, and starch, were added and thoroughly blended. Distilled water and soybean oil containing dissolved cholesterol were then incorporated, and the mixture homogenized again. The final mixture was extruded into 3 mm diameter pellets using a feed extruder. The prepared diets were air-dried, sealed in plastic bags, and stored at −20°C until use.

### 2.2. Experimental Fish

All experimental protocols involving fish were conducted in compliance with the ethical guidelines outlined in the National Institutes of Health's Guide for the Care and Use of Laboratory Animals (NIH Publication No. 8023, revised 1978) and received formal approval from the Shantou University's Institutional Animal Care and Use Committee (Guangdong, China). Juvenile Nile tilapia were sourced from Guangzhou Tianfa Aquatic Development Co., Ltd. (Guangdong, China), and underwent a 2-week acclimation period in an indoor freshwater recirculating aquaculture system during which time they were fed a commercial tilapia feed. Following acclimation, 180 healthy fish with comparable initial body weights (1.79 ± 0.04 g) were distributed randomly into six tanks (0.4 m × 0.6 m × 0.6 m) with 30 fish per tank (90 fish per dietary treatment). Three tanks were assigned at random to each diet to form two experimental groups: the C and HC groups. During the 8-week feeding trial, fish were fed twice daily at a rate equivalent to 4% of the total body weight per day. Weekly bulk weighing of fish in each tank allowed for adjustments to the feeding rations. Water temperature was kept at 26.0 ± 2.0°C. Total ammonia–nitrogen levels were maintained below 0.25 mg/L, while pH levels ranged from 7.6 to 8.1, and dissolved oxygen levels ranged from 5.1 to 6.6 mg/L.

### 2.3. Sample Collection

After the 8-week feeding trial, the fish were fasted overnight before being sampled. Four fish per tank (12 fish per dietary treatment) were selected at random and euthanized by an overdose of anesthetic (MS-222: tricaine methanesulfonate, Sigma, USA; 60–80 mg/L). All sampled fish were weighed individually for determination of growth performance parameters. Blood was collected from the tail vein into heparinized tubes, and plasma was obtained after centrifugation at 4000 *g* for 10 min for analysis of plasma biochemistry. The weights of the carcass, liver, and visceral mass of each fish were recorded to calculate the carcass ratio (CR), hepatosomatic index (HSI) and viscerosomatic index (VSI), respectively. Liver, intestine, spleen, and head kidney were collected from each fish, and three separate samples were dissected from each tissue and immediately snap-frozen in liquid nitrogen, followed by storage at −80°C for subsequent analyses of biochemical composition, enzyme activities, and gene expression. For liver, a fourth sample (3 mm × 3 mm × 3 mm) was dissected and fixed in 4% paraformaldehyde solution or electron microscope section fixative for subsequent histological analysis.

### 2.4. Biochemical Parameters

All assays were performed using commercial assay kits (Jiancheng Biotech Co., Nanjing, China). Total cholesterol (TC; Kit A111-1-1) levels were measured using the cholesterol oxidase method, where cholesterol is oxidized by cholesterol oxidase to produce H_2_O_2_, which then reacts with 4-aminoantipyrine and phenol under peroxidase catalysis to form a red quinoneimine compound. The absorbance of this product, proportional to TC concentration, is quantified spectrophotometrically Superoxide dismutase (SOD; A001-3-2) activity was determined via the xanthine oxidase method, which involves the superoxide anion generated by the xanthine–xanthine oxidase system reducing nitroblue tetrazolium (NBT) to formazan. SOD inhibits this reduction, and the degree of inhibition reflects its activity. Catalase (CAT; A007-1-1) activity was assessed by its ability to decompose hydrogen peroxide (H_2_O_2_), and H_2_O_2_ remaining reacts with ammonium molybdate to form a yellow complex, with absorbance inversely proportional to CAT activity. Malondialdehyde (MDA, A003-1-1) was quantified using the thiobarbituric acid (TBA) method, where MDA reacts with TBA under acidic conditions to generate a red adduct, measured at 532 nm. Serum alanine aminotransferase (ALT; C009-2-1) and aspartate aminotransferase (AST; C010-2-1) activities were determined by rate assays, wherein ALT catalyzes the transfer of an amino group from alanine to α-ketoglutarate, producing pyruvate and glutamate, while AST transfers an amino group from aspartate to α-ketoglutarate, forming oxaloacetate and glutamate. The coupled oxidation of NADH to NAD^+^, mediated by lactate dehydrogenase (for ALT) or malate dehydrogenase (for AST), is monitored via absorbance decline at 340 nm, correlating with enzyme activity. All assays were performed following the manufacturer's standardized protocols (Jiancheng Biotech Co.), with absorbance measurements conducted using a MultiMode Microplate Reader (SpectraMax i3x, Thermo Fisher Scientific).

### 2.5. Liver Histology

Hematoxylin and eosin (H&E): liver tissue samples were fixed in 4% paraformaldehyde solution for 48 h before the specimens were dehydrated through a graded ethanol series, cleared in xylene, and embedded in paraffin wax. Sections with a thickness of 5 μm were cut with a microtome and stained with H&E. Stained sections were observed under a light microscope (Axio Imager 2, Zeiss, Germany), and images were captured by a camera (Axiocam 506, Zeiss) histological images obtained were captured and analyzed using CaseViewer software (Servicebio Biotechnology Co., Ltd, Wuhan, China).

Transmission electron microscopy (TEM): liver samples were fixed in glutaraldehyde and osmium tetroxide, dehydrated through an acetone series, and embedded in epoxy resin. Ultrathin sections (70 nm) were cut and stained with uranyl acetate/lead citrate prior to observation using a Hitachi HT7700 TEM. Methods followed established laboratory protocols [9].

### 2.6. Real-Time Quantitative PCR Analysis

Liver and head kidney tissues were collected for total RNA extraction using Trizol reagent (Takara, Japan) following the manufacturer's instructions. The purity of the extracted total RNA was evaluated by measuring A260/A280 and A260/230 ratios using spectrophotometry (NanoDrop 2000, Thermo Scientific, San Jose, CA, USA), with acceptable ranges of 1.8–2.0 and >2.0, respectively. Additionally, RNA integrity was verified by analysis of 28S/18S rRNA bands via 1% agarose gel electrophoresis. Reverse transcription was performed using the HiScript II 1st Strand cDNA Synthesis Kit (Vazyme, Nanjing, China), which included a step for genomic DNA elimination. Then, real-time quantitative PCR was carried out on a CFX Connect Real-Time System (Bio-Rad, China) using Ultra SYBR Mixture (Aidlab, China) following the corresponding quantification program. The primer sequences for both reference genes (β-actin and 18S ribosomal) and target genes are listed in Table 2. Standard curves were established for all target genes, demonstrating excellent linearity (*R*^2^ > 0.99), and amplification efficiencies (90%–110%), calculated from the linear regression of log_10_ (template dilution) vs. Ct values, confirmed optimal PCR performance. The relative expression levels of target genes were calculated using the 2^−∆∆Ct^ method [19].

### 2.7. Transcriptome Analysis of Liver

Briefly, RNA-seq data were generated using the HiSeq3000 platform. Bioinformatics tools were employed to perform quality control on the raw data, removing low-quality reads, and adapter sequences to ensure data accuracy and reliability. The resulting clean data were aligned to a reference genome for transcript assembly and quantification, enabling the determination of gene expression levels. Differentially expressed genes under various treatments were identified based on thresholds: |log_2_FC| ≥ 1 and *p* < 0.05. To further explore the functions of these genes, functional annotation and enrichment analyses were conducted. These included Gene Ontology (GO) annotation and Kyoto Encyclopedia of Genes and Genomes (KEGG) pathway enrichment analysis, providing insights into the biological processes and signaling pathways associated with the differentially expressed genes, as demonstrated previously [9].

### 2.8. Metabolome Analysis

For metabolomic profiling, liver tissue was processed using the XploreMET platform (Metabo-Profile, China) as described previously [20], with modifications. Briefly, 50 mg tissue was homogenized in 80% methanol/water (4:1, v/v) containing 0.1% formic acid and internal standards (2-chloro-L-phenylalanine, lidocaine), followed by centrifugation (14,000 × *g*, 15 min, 4°C). Metabolite separation was achieved using an Agilent 1290 Infinity II UHPLC system equipped with a ZORBAX Eclipse Plus C18 column (2.1 mm × 100 mm, 1.8 μm) using a gradient of 0.1% formic acid in water and acetonitrile at 0.4 mL/min. Mass spectrometry was performed on an Agilent 6545xT Q-TOF mass spectrometer operated in ESI positive/negative modes (m/z 50–1000, capillary voltage 3500 V). Raw data were processed through Progenesis QI (v3.0, Waters) with quality filters (peak intensity > 10,000 counts; QC CV < 30%) and identified against HMDB/METLIN databases. Differential metabolites were defined by OPLS-DA (SIMCA 16.0) with VIP > 1.0, |log_2_FC| ≥ 1, and *p* < 0.05 (Student's *t*-test), following established metabolomics standards.

### 2.9. Statistical Analysis

Data are presented as means ± standard deviation (SD) with *n* = 3 (i.e., tank replicates) for growth and transcriptomic analysis. However, morphological data were presented as *n* = 12 (individual fish) and biochemical, enzyme activity, and gene expression data are presented as *n* = 6 (two pools of two fish per tank). Before conducting statistical analysis, all data were tested for homogeneity of variances using Levene's test. Subsequently, a Student's *t*-test was performed between the C and HC groups with a significance threshold (*p* < 0.05). All analyses were carried out using SPSS Statistics 23.0 software (IBM, Michigan Avenue, USA).

## 3. Results

### 3.1. High Cholesterol Intake Caused Obvious Lipid Peroxidation in the Liver and Head Kidney of Fish

**Figure 1:**
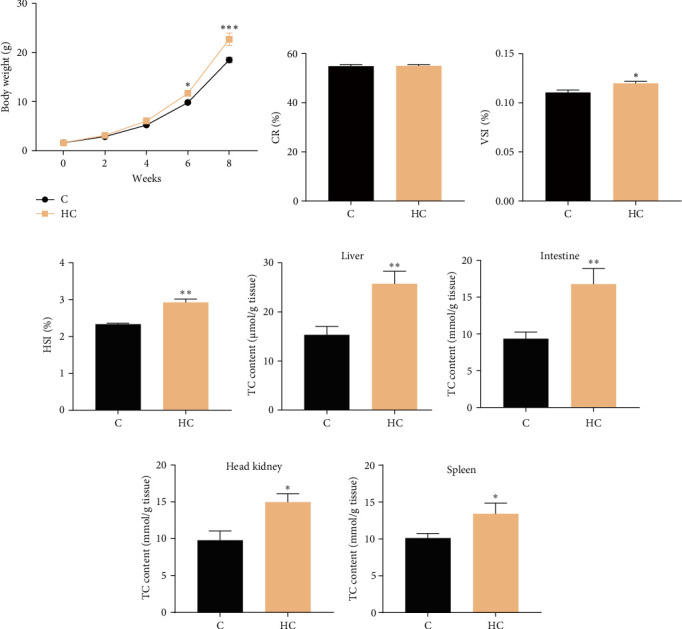
Effects of HC diet on growth, organosomatic indices, and total cholesterol contents of various tissues of Nile tilapia. (A) Body weight. (B) Carcass ratio (CR). (C) Viscerosomatic index (VSI). (D) Hepatosomatic index (HSI). (E–H) Total cholesterol (TC) contents of liver, intestine, head kidney, and spleen. Data are presented as means ± SD (*n* = 3 tanks/treatments for body weight; *n* = 12 individual fish/treatment for organosomatic indices; *n* = 6 individual fish/treatment for cholesterol contents of tissues). Asterisks on bars indicate significant differences (*⁣*^*∗*^*p* < 0.05, *⁣*^*∗∗*^*p* < 0.01, *⁣*^*∗∗∗*^*p* < 0.001) between C and HC groups. C, control diet; HC, high cholesterol diet.

**Figure 2:**
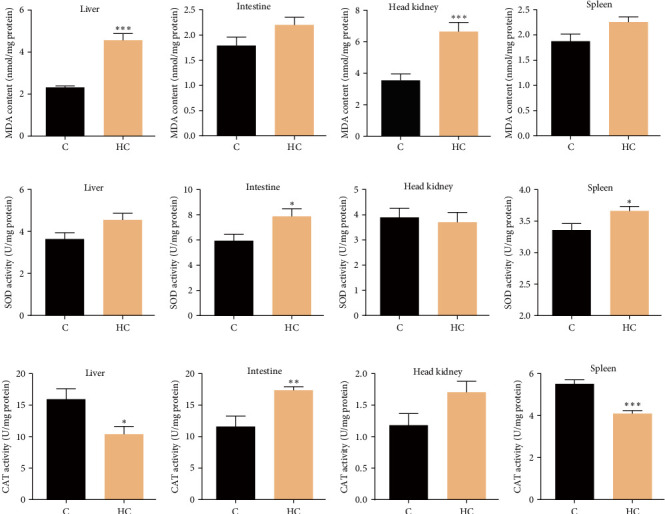
Effects of HC diet on MDA contents, and SOD and CAT enzyme activities in different tissues of Nile tilapia. (A–D) Malondialdehyde (MDA) contents of liver, intestine, head kidney, and spleen. (E–H) Superoxide dismutase (SOD) activities in liver, intestine, head kidney, and spleen. (I–L) Catalase (CAT) activities in liver, intestine, head kidney, and spleen. Data are presented as means ± SD (*n* = 6 individual fish). Asterisks on bars indicate significant differences (*⁣*^*∗*^*p* < 0.05, *⁣*^*∗∗*^*p* < 0.01, *⁣*^*∗∗∗*^*p* < 0.001) between C and HC groups. C, control diet; HC, high cholesterol diet.

After the 8-week feeding trial, fish body weight, VSI, and HSI were increased significantly in tilapia fed HC (*p* < 0.05; Figure 1A,C,D), but the CR showed no obvious change between the C and HC groups (Figure 1B). Additionally, HC intake caused noticeable cholesterol accumulation in the liver (increased by 67.9% in HC compared to C, *p* < 0.01), intestine (79.6%, *p* < 0.01), head kidney (50.6%, *p* < 0.05), and spleen (38.0%, *p* < 0.05) (Figure 1E–H). Elevated levels of MDA were detected in the liver (67.9%, *p* < 0.001) and head kidney (87.7%, *p* < 0.001) of fish fed HC (Figure 2A,C), while no significant alteration was observed in the intestine and spleen (*p* > 0.05; Figure 2B,D). The HC diet had no significant effects on activities of SOD in liver and head kidney and CAT in head kidney (Figure 2E,G,K). In contrast, SOD enzyme activities increased significantly in intestine and spleen (32.5% and 9.2%, respectively; *p* < 0.05; Figure 2F,H), while CAT activity also increased significantly in intestine (34.8%, *p* < 0.05; Figure 2J) but decreased significantly in liver and spleen (34.8% and 25.6%, respectively; *p* < 0.05; Figure 2I,L) in fish fed the HC diet. These results suggested that intestine exhibited a higher antioxidant capacity to protect against the stress of cholesterol accumulation, while liver, head kidney showed no increased antioxidant function that could lead to oxidative stress and lipid peroxidation.

### 3.2. High Cholesterol Intake Caused ER Stress, Inflammation, and Apoptosis in the Liver of Fish

**Figure 3:**
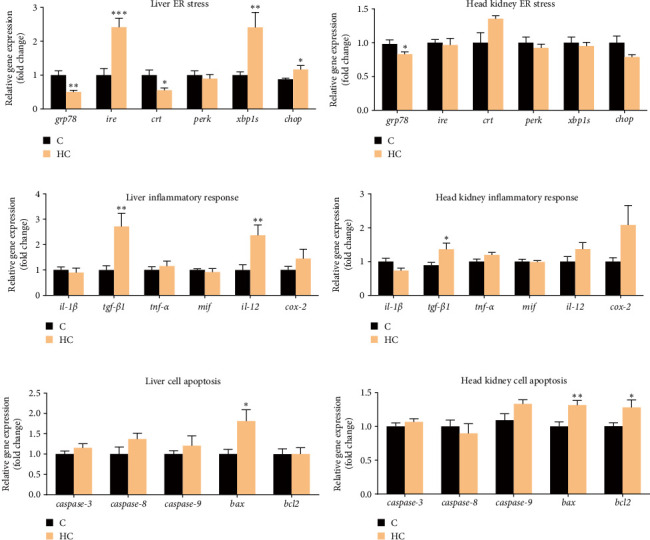
Effects of HC diet on ER stress, inflammation, and apoptosis in liver and head kidney of Nile tilapia. (A, B) expression of genes associated with endoplasmic reticulum (ER) stress in liver and head kidney. (C, D) Expression of genes associated with inflammatory response in liver and head kidney. (E, F) Expression of genes associated with cell apoptosis in liver and head kidney. Data are presented as means ± SD (*n* = 6 individual fish). Asterisks on bars indicate significant differences (⁣^*∗*^*p* < 0.05, ⁣^*∗∗*^*p* < 0.01, ⁣^*∗∗∗*^*p* < 0.001) between C and HC groups. C, control diet; HC, high cholesterol diet; *grp78*: glucose regulated protein, 78kDa; *ire*: inositol requiring enzyme; *crt*: calreticulin; *perk*: protein kinase like endoplasmic reticulum kinase; *xbp1s*：X box binding protein 1; *chop*: C/EBP homologous protein; *il-1β*: interleukin 1 beta; *tgf-β1*: transforming growth factor beta 1; *tnf-α*: tumor necrosis factor a; *mif*: migration inhibitory factor; *il-12*: interleukin 12; *cox-2*: *ptgs2*, prostaglandin endoperoxide synthase 2; *casp3/8/9*: caspase 3/8/9 apoptosis related cysteine peptidase; *bax*: B cell lymphoma 2 associated X; *bcl2*: B cell lymphoma 2.

Expression levels of genes related to antioxidation, ER stress, inflammatory response, and apoptosis were determined in the liver and head kidney of tilapia. The results showed that HC intake significantly upregulated expression levels of ER stress-related genes, X-box binding protein 1 (*xbp1s*), inositol requiring enzyme (*ire*), and C/EBP homologous protein (*chop*), but significantly downregulated expression levels of glucose-regulated protein, 78kDa (*grp78*), and calreticulin (*crt*) genes in liver (*p* < 0.05; Figure 3A). Additionally, HC intake significantly increased expression levels of genes related to inflammatory responses, such as transforming growth factor β1 (*tgf-β1*) and interleukin 12 (*il-12*), and the expression of apoptosis-related gene B-cell lymphoma2-associated X (*bax*) in liver (*p* < 0.05; Figure 3C,E). In the head kidney, there were no significant changes in the expression of ER stress-related genes in fish fed the HC diet (*p* > 0.05; Figure 3B). Although expression levels of genes related to the inflammatory response and apoptosis, such as *tgf-β1* and *bax*, were increased significantly, expression of the anti-apoptosis-related gene B-cell lymphoma·2 (*bcl2*) was also upregulated significantly in head kidney of fish fed HC diet (*p* < 0.05; Figure 3D,F). Overall, cholesterol accumulation caused ER stress, inflammation, and apoptosis in the liver, while these effects were less pronounced in the head kidney.

### 3.3. High Cholesterol Intake Inhibited Liver Glutathione Metabolism and Tricarboxylic Acid Cycle

**Figure 4:**
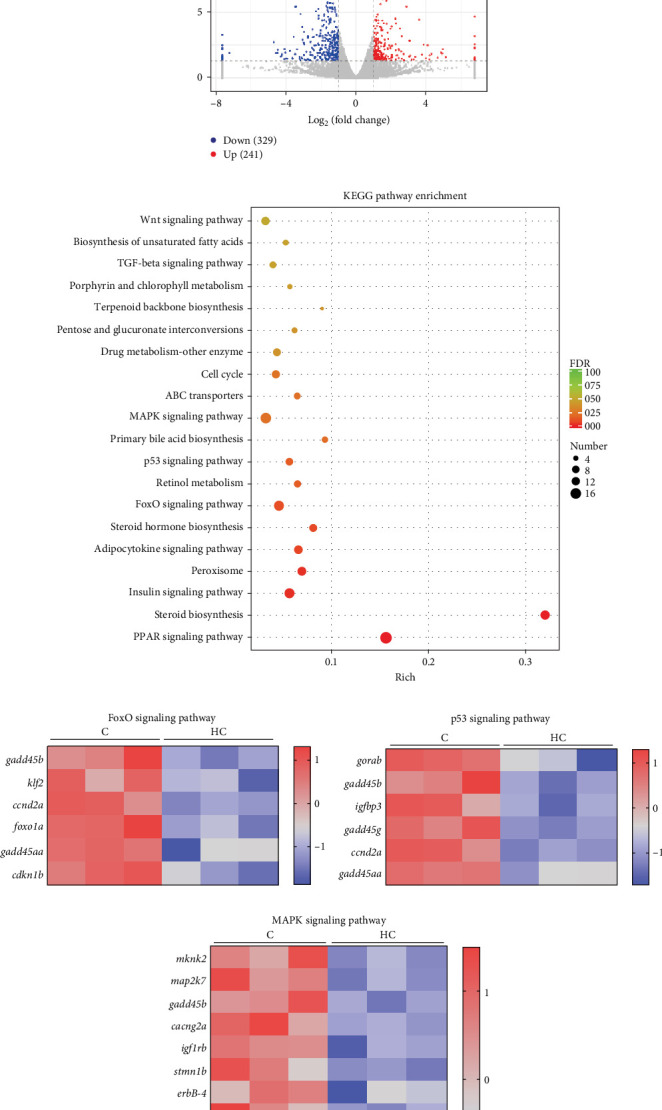
Transcriptome analysis of differentially expressed genes in the liver of Nile tilapia fed HC diet. (A) Volcano plots. (B) KEGG pathway analysis of differentially expressed genes; (C–E) differentially expressed genes related to FoxO, p53, and MAPK signaling pathway. Data presented are based on tank means (*n* = 3). C: control diet; HC: high cholesterol diet; *gadd45b*: growth arrest and DNA damage inducible 45b; *klf2*: kruppel like factor 2; *ccnd2a*: cyclin D 2a; *foxo1a*: forkhead box O1A; *gadd45a*: growth arrest and DNA damage inducible 45a; *cdkn1b*: clin dependent kinase inhibitor 1b; *gorab*: RAB6 interacting golgin; *igfbp3*: insulin like growth factor binding protein 3; *gadd45g*: growth arrest and DNA damage inducible 45g; *mknk2*: interacting serine/threonine kinase 2; *map2k7*: mitogen activated protein kinase kinase 7; *cacng2a*: calcium voltage gated channel auxiliary subunit gamma 2a; *igf1rb*: insulin like growth factor 1 receptor b; *stmn1b*: sapiens stathmin 1b; *erbB4*: erb-b2 receptor tyrosine kinase 4; *myc*: musculus myelocytomatosis oncogene; *dusp1*: dual specificity phosphatase 1; *hgf*: hepatocyte growth factor; *rbsk6a2*: ribosomal protein S6 kinase a2; *nr4a1*: nuclear receptor subfamily 4 group A member 1.

**Figure 5:**
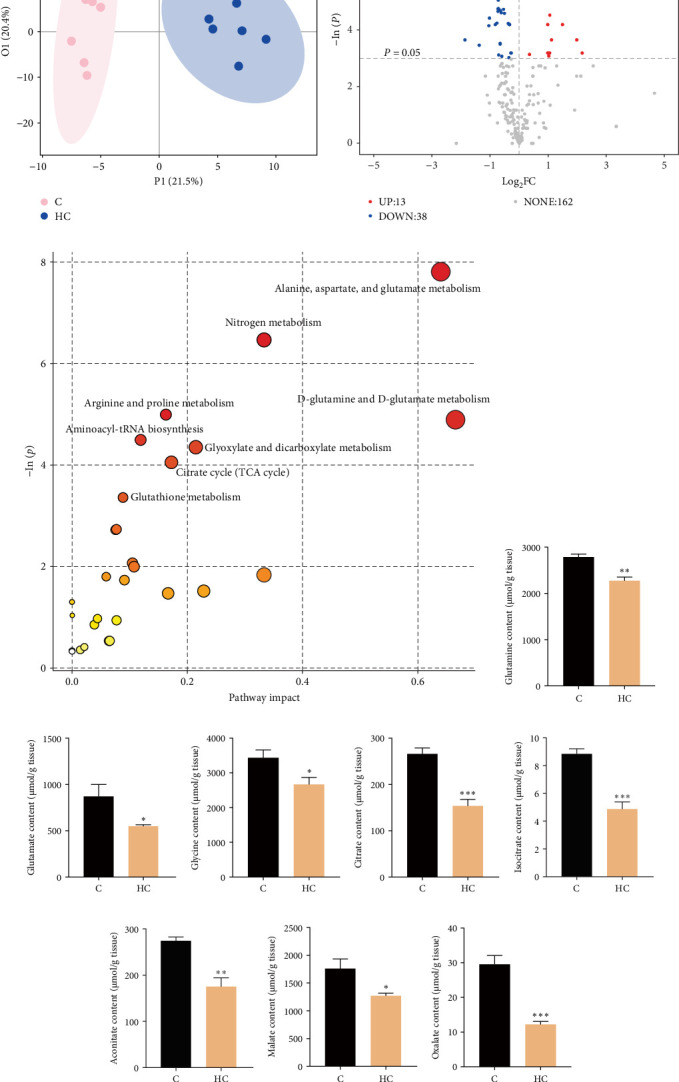
The metabolite profiles in the liver of Nile tilapia fed HC diet. (A) Partial least squares-discriminant analysis (PLS-DA). (B) Volcano plot for identifying differential metabolites. (C) Differential metabolite enrichment analysis. (D–F) The contents of glutamine, glutamate, and glycine in liver. (G–K) The contents of tricarboxylic acid cycle intermediate metabolites: citrate, isocitrate, aconitate, malate, and oxalate in liver. Data are presented as means ± SD (*n* = 6 individual fish). Asterisks on bars indicate significant differences (*⁣*^*∗*^*p* < 0.05, *⁣*^*∗∗*^*p* < 0.01, *⁣*^*∗∗∗*^*p* < 0.001) between C and HC groups. C, control diet; HC, high cholesterol diet.

The differences in transcriptional expression profiles in the liver of fish fed the C and HC diets were further analyzed and the results showed distinct sample clustering between the groups, with 241 genes significantly upregulated and 329 genes downregulated (Figure 4A). KEGG enrichment analysis showed the involvement of mitogen-activated protein kinases (MAPKs), Forkhead box O (FoxO), and p53 signaling pathways in responses to environmental stress and apoptosis processes (Figure 4B–E). Additionally, metabolome analysis distinctly clustered the C and HC groups, revealing 38 metabolites with decreased contents and 13 metabolites with increased contents in the liver of tilapia fed the HC diet compared to fish fed the C diet (Figure 5A,B). Moreover, differences in metabolite levels were enriched primarily in amino acid metabolism, including glutamine and glutamate, as well as glutathione metabolism, with significant reductions observed in glutamine (18.4%, *p* < 0.01), glutamate (37.3%, *p* < 0.05), and glycine (22.1%, *p* < 0.05) contents (Figure 5C–F). Furthermore, enrichment of tricarboxylic acid cycle (TCA) metabolites was observed, with significant reductions in key intermediates including citrate (42.5%, *p* < 0.001), isocitrate (44.9%, *p* < 0.001), aconitate (26.0%, *p* < 0.01), malate (27.9%, *p* < 0.05), and oxalate (59.0%, *p* < 0.001) noted in the liver of fish fed HC diet (Figure 5G–K). These results indicated that cholesterol accumulation led to significant alterations in metabolic pathways, including reduced glutathione metabolism and the TCA cycle.

### 3.4. High Cholesterol Intake Triggered Liver and Mitochondria Damage in Fish

**Figure 6:**
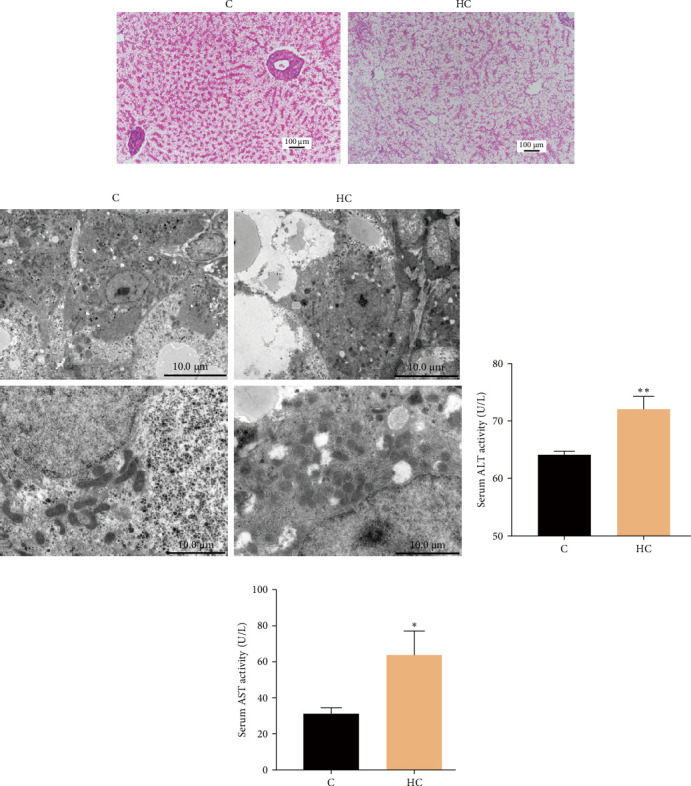
Effects of HC diet on liver health of Nile tilapia. (A) Hematoxylin and Eosin (H&E) staining of the liver, scale bars, 100 μm. (B) Transmission electron microscope (TEM) images of liver. (C, D) Serum alanine aminotransferase (ALT) and aspartate aminotransferase (AST) enzymes activities. Data are presented as means ± SD (*n* = 3 individual fish for histology and *n* = 6 individual fish for enzyme activities. Asterisks on bars indicate significant differences (*⁣*^*∗*^*p* < 0.05, *⁣*^*∗∗*^*p* < 0.01) between C and HC groups. C, control diet; HC, high cholesterol diet.

Histological analysis showed that HC intake caused obvious hepatic cellular vacuolation and notable damage to the central vein (Figure 6A). Analysis by TEM showed conspicuous accumulation of lipid droplets within hepatocytes and distinct alterations in the morphology of mitochondria in fish fed the HC diet (Figure 6B). Moreover, HC intake significantly increased serum AST and ALT activities by 106.7% and 12.4%, respectively (*p* < 0.05; Figure 6C,D). These findings collectively suggested that cholesterol accumulation impaired mitochondrial function and liver health in tilapia fed the HC diet.

## 4. Discussion

### 4.1. High Cholesterol Intake Induced Oxidative Stress Associated With Compromised Antioxidant and Mitochondrial Function

Cholesterol can be derived from the diet or synthesized endogenously and originates primarily from the latter in many animals. Fish were thought to grow healthily without requiring dietary cholesterol [21], due to them possessing cholesterol synthesis pathways similar to mammals, which allow regulation in response to cellular cholesterol levels and requirements [22, 23]. Animal-derived ingredients like fishmeal and fish oil possess low levels of cholesterol content, whereas plant-based ingredients such as soybean meal and oil are essentially devoid of cholesterol. This distinction could potentially compromise fish growth and health when fed a plant-based diet. Indeed, supplementation of 1.5% cholesterol to soybean meal-based diets showed growth-promoting effects in channel catfish and turbot [6, 7]. However, elevated cholesterol levels in serum and liver were observed in channel catfish, raising concerns about potential health implications [7]. It is noteworthy that cholesterol accumulation has often been documented in fish fed high-fat or high-carbohydrate diets, often accompanied by metabolic disorders and health impairment [2, 24]. Moreover, it was reported that dietary cholesterol supplementation increased the occurrence of fatty liver and arteriosclerotic lesions in fish [8]. Our recent study also revealed that cholesterol accumulation caused metabolic disorders and health damage in tilapia [9]. In the present study, HC intake significantly increased fish body weight, although CR was unaffected. However, increased VSI and HSI suggested possible lipid accumulation in the visceral organs of fish fed HC diet, and further analysis confirmed that HC intake caused accumulation of cholesterol in the liver, intestine, head kidney, and spleen of tilapia, and this might potentially damage fish health.

In mammals, it was observed that rat hepatic stellate cells exhibited higher susceptibility to oxidative stress, along with mitochondrial dysfunction and apoptosis, induced by HC treatment. This suggested that cholesterol could potentially serve as a catalyst for lipid peroxidation and cellular death in hepatic stellate cells of a nonalcoholic steatohepatitis model [25]. Among the numerous biological targets affected by oxidative stress, lipids emerge as the primary class of biomolecules involved. The process of lipid oxidation yields a variety of secondary products, with MDA resulting from the peroxidation of polyunsaturated fatty acids, the most extensively investigated. This aldehyde compound exhibits considerable toxicity, highlighting its importance beyond simply serving as an indicator of lipid peroxidation [26]. A study in hepatocellular carcinoma showed previously that excessive cellular cholesterol caused the accumulation of lipid peroxides [27]. In the present study, HC intake generally elevated the contents of MDA in tissues but, especially, the liver and head kidney, suggesting that lipid peroxidation was triggered by the accumulation of cholesterol in these tissues. The primary defense antioxidant defense enzymes, SOD and CAT, are pivotal within the broader antioxidant defense system having important roles in neutralizing free radicals, byproducts constantly generated during normal metabolic processes, including the mitochondrial electron transport chain [28]. Indeed, a previous study showed that cholesterol accumulation impaired antioxidant capacity by inhibiting the activities of CAT and SOD enzymes in Chinese hamster ovary (CHO) cells [29]. In the present study, the activity of SOD was generally increased in tissues, whereas CAT showed a more variable pattern but was increased in the intestine. Overall the results suggested that the intestine exhibited a higher antioxidant capacity to protect from the stress of cholesterol accumulation, while the liver, head kidney, and perhaps spleen may be more at risk from the damaging effects of free radicals, consistent with the lipid peroxidation observed in the liver and head kidney under conditions of cholesterol accumulation.

Cholesterol plays a pivotal role in modulating the structural and functional properties of the lipid bilayer in all cellular membranes bilayers, including those of mitochondria. Normally the cholesterol content in mitochondria is relatively low, but when cholesterol accumulates beyond physiological levels it, adversely affects mitochondrial function through impairing the glutathione redox cycle and assembly of respiratory supercomplexes, thereby increasing generation of ROS [10]. Glutathione (GSH), a tripeptide molecule consisting of cysteine, glycine, and glutamic acid, is a crucial component of the antioxidant system [30, 31]. In a mouse model of hepatic steatosis, cholesterol accumulated in mitochondria leading to reduced fluidity of the inner mitochondrial membrane and consequent depletion of mitochondrial GSH. Studies have reported that dicarboxylate carrier (DIC) and 2-oxoglutarate carrier (OGC) are involved in the transport of GSH from the cytosol into mitochondria. Excessive intracellular cholesterol accumulation may impair the GSH-transporting capacity of DIC and OGC by compromising the fluidity of the inner mitochondrial membrane [10]. In the present study, metabolome analysis showed that the differences in metabolite levels between tilapia fed the C and HC diets were enriched in amino acid metabolism, including glutamine and glutamate, as well as glutathione metabolism, with significant reductions in glutamine, glutamate, and glycine content. This suggested that cholesterol accumulation also led to notable alterations in metabolic pathways, including the inhibition of glutathione metabolism, resulting in a compromised antioxidant defense system. Additionally, it should be noted that mitochondria are the primary cellular source of ROS, with enzymes in the electron transport chain notably contributing to this oxidative phenomenon [32]. Previously, a study reported that cholesterol treatment damaged mitochondrial structure and function, leading to intracellular oxidative damage in human regulatory T cells [13]. In high-fat diet mouse models, mitochondrial dysfunction manifested earlier than alterations in blood levels of TCA cycle metabolites [33]. Given that the TCA cycle occurs within the mitochondrial matrix, these two processes are physiologically interconnected. Evidence indicates that the reduction in the TCA cycle intermediates is a direct consequence of mitochondrial structural and functional impairment. Thus, the cholesterol-induced reduction in mitochondrial membrane fluidity initiates a self-perpetuating loop with structural damage impairing metabolite transporters, disrupting the TCA cycle, and promoting ROS bursts, which further exacerbate mitochondrial dysfunction. In the present study, HC intake caused accumulation of lipid droplets within hepatocytes and distinct alterations in the morphology of mitochondria in tilapia. Moreover, metabolome analysis indicated that differences in metabolite levels were mainly enriched in TCA cycle, with reduced intermediate metabolites in the liver of fish fed the HC diet. This suggested that cholesterol accumulation impaired mitochondrial structure and function in the liver of tilapia fed HC. Overall, this collectively indicated that the oxidative stress induced by cholesterol accumulation might be associated with reduced antioxidant capability and impaired mitochondrial function.

### 4.2. High Cholesterol Intake Caused ER Stress, Inflammation, and Apoptosis in Fish Liver

In animals, oxidative stress and ER stress are closely interrelated phenomena, as oxidative stress has the capacity to induce protein misfolding by perturbing the ER redox state and interfering with disulfide bond formation, thereby exacerbating protein misfolding processes [34]. The unfolded protein response (UPR) acts as a supplementary adaptive mechanism in response to ER stress. It is orchestrated by three ER transmembrane receptor proteins: activating transcription factor 6 (ATF6), IRE1, and protein kinase RNA-like ER kinase (PERK). Under normal conditions, the molecular chaperone, GRP78, binds to these receptors, maintaining them in an inactive state. However, during ER stress, the accumulation of URP triggers the dissociation of GRP78 from ATF6, IRE1, and PERK, resulting in their activation [35]. Previous research found that HC treatment induced ER stress and enhanced the expression of CHOP, which is a protein involved in ER stress-induced apoptosis. Mechanistically, the accumulation of cholesterol in hepatocytes activated the IRE1/p38 branch of ER stress, leading to elevated levels of CHOP [36]. In the present study, the expression levels of ER stress-related genes, such as *ire*, *xbp1s*, and *chop* were increased, but the expression levels of *grp78* and *crt* genes were decreased in the liver of fish fed the HC diet. Moreover, ER stress triggered the activation of cytokines implicated in inflammatory responses via resident sensors of the UPR and signaling proteins within toll-like receptor (TLR) pathways [37]. Additionally, when oxidative stress exceeds the threshold of this defense capability, cells would transition to apoptotic pathways to initiate cell death [15, 16]. Accordingly, previous studies reported a positive correlation between cholesterol content and inflammatory levels in the liver of mice [38], and that cholesterol accumulation led to oxidative stress and cell apoptotic in CHO cells [29]. In the present study, HC intake increased inflammatory responses and cell apoptosis in the liver of tilapia. Furthermore, KEGG pathway enrichment analysis of the liver transcriptome indicated the involvement of FoxO, p53, and MAPK pathways in responses to environmental stress and apoptosis processes. Moreover, a previous study showed that guinea pigs fed a HC diet exhibited higher serum ALT and AST activities, which are indicative of liver injury. Additionally, the percentage distribution of cholesterol exhibited positive correlations with serum ALT and AST activities, suggesting a direct relationship between increased cholesterol and elevated markers of liver damage [39]. In the present study, high intake of cholesterol elevated serum ALT and AST enzyme activities and caused hepatic cellular vacuolation and destruction of the central vein indicating that cholesterol accumulation caused liver damage in tilapia fed the HC diet. Overall, the liver exhibited more sensitivity to HC intake, leading to oxidative stress and ultimately resulting in health damage in Nile tilapia. The observed histopathological changes, including vacuolation, vascular damage, apoptosis, and mitochondrial swelling/fragmentation, reflected the underlying biochemical cascade triggered by cholesterol overload. This sequence progressed from mitochondrial and ER stress to ROS/GSH imbalance, driving lipid peroxidation, inflammatory signaling, and ultimately apoptotic cell death and tissue necrosis.

## 5. Conclusions

The present study showed that cholesterol accumulation induced lipid peroxidation and health damage in the liver of Nile tilapia fed the HC diet. These adverse effects were linked to the oxidative stress, which subsequently triggered ER stress, inflammation, and apoptosis in the liver. The oxidative stress induced by cholesterol accumulation might be associated with impaired antioxidant and mitochondrial function. Therefore, lowering cholesterol accumulation could be an effective strategy for potentially alleviating liver damage induced by metabolic syndrome diseases in aquatic animals. However, key mechanistic and therapeutic questions remain, particularly regarding mitochondrial glutathione recycling and respiratory supercomplex stabilization to counteract ROS overproduction. From an aquaculture perspective, dietary cholesterol in tilapia diets should be maintained below 1.0% to balance metabolic safety with growth benefits. In addition, functional dietary additives such as coenzyme Q10 or the bile acid tauroursodeoxycholic acid may help mitigate negative effects in cholesterol-overloaded fish. However, given the distinctive lipid metabolism of tilapia, further validation in other farmed fish (e.g., salmonids) is required before wider application.

## Figures and Tables

**Table 1:** Formulations and proximate compositions of the experimental diets.

Ingredients (g/kg)	Control (C)	High cholesterol (HC)
Casein	320	320
Gelatin	90	90
Corn starch	320	320
Soybean oil	60	60
Vitamin^a^	15	15
Minerals^b^	25	25
CMC	24.75	24.75
Cellulose	130	114
Choline chloride	5	5
Ca(H_2_PO_4_)_2_	10	10
Dimethly-β-propiothetin	0.25	0.25
Cholesterol	0	16
Proximate composition (%, dry matter)		
Dry matter (% wet wt.)	90.2	91.2
Crude protein	38.5	38.1
Crude lipid	6.1	7.5
Cholesterol	0.01	1.5
Ash	5.8	6.2

^a^Vitamin premix (mg or IU/kg): 500,000 I.U. (international units): vitamin A; 50,000 I.U: vitamin D3; 2500 mg: vitamin E; 1000 mg: vitamin K3; 5000 mg: vitamin B1; 5000 mg: vitamin B2; 5000 mg: vitamin B6; 5000 μg: vitamin B12; 25,000 mg: inositol; 10,000 mg: pantothenic acid; 100,000 mg: choline; 25,000 mg: niacin; 1000 mg: folic acid; 250 mg: biotin; 10,000 mg: vitamin C.

^b^Mineral premix (g/kg): 314.0 g, CaCO_3_; 469.3 g, KH_2_PO_4_; 147.4 g, MgSO_4_·7H_2_O; 49.8 g, NaCl; 10.9 g, Fe(II) gluconate; 3.12 g, MnSO_4_·H_2_O; 4.67 g, ZnSO_4_·7H_2_O; 0.62 g, CuSO_4_·5H_2_O; 0.16 g; 0.08 g, CoCl_2_·6H_2_O; 0.06 g, NH_4_ molybdate; 0.02 g, NaSeO_3_.

**Table 2:** The primer sequences used for quantitative real-time PCR analysis in Nile tilapia.

Gene	Forward primer (5'- to 3'-)	Reverse primer (5'- to 3'-)	Amplification efficiency (%)	Accession no.
*grp78*	GCAAGCCCCATATCCAGGTT	CCAGGTAAGCCTCAGCAGTC	94	XM_019357245.1
*caspase 3*	GGAGTGGACGATACAGACGCAAA	TGAAGCTGTGTGACTGGGGCTT	93	NM_001282894.1
*caspase 8*	AACTGGAATCTTCTGAGGTGGCA	TTGAGAAGAGATCTTTGGCCTGC	95	XM_005475926.3
*caspase 9*	ATACTTGAGGAAAACGCTGCCACT	GAACCAGGCATTTGTTTGTAGAGC	96	XM_003455320.4
*bax*	GGAGGAGGCGATCAAGGGAAT	TTTTTGCCTCTGAACTCGCTCA	99	XM_019357746.1
*bcl2*	ATCGCAGACTGGATGACGGAGTAT	TCTGTCTGTCGTACAGCTCCACAA	97	XM_003437902.4
*ire*	TGTAAGAAGCTCGCAGTGGG	TTGTCTTTGCAGTCCTCGCT	95	XM_005461083.3
*crt*	GATGCCAAGAAGCCCGATGA	GGCAGGGTTGTCGATCTCTC	102	XM_005478351.4
*perk*	GCAGAGTCCAGCGTTTACCT	CGATGGCTTTGGAGGGGAAT	94	XM_003447769.4
*xbp1s*	GCCTTTCCTGTTACGGAGGTTGTG	GCCAGGCAGGCTTCTTTCTCC	98	XM_031746876.2
*chop*	TACATGCACCGAGAAGGAGC	GACGAGTTGTGATGCAGGGT	104	XM_003451826.5
*il-1β*	GAGCACAGAATTCCAGGATGAAAG	TGAACTGAGGTGGTCCAGCTGT	95	XM_019365841.1
*tgf-β1*	AAGAGGAGGAGGAATACTTTGCCA	GAAGCTCATTGAGATGACTTTGGG	96	NM_001311325.1
*tnf-α*	CAGAAGCACTAAAGGCGAAGAACA	TTCTAGATGGATGGCTGCCTTG	94	NM_001279533
*mif*	AGCAGAAGCAGGAAGGCGAAGA	CGGTACATCACCTCTGGCAACATT	102	XM_003444573.3
*il-12*	AGGTCAGCCAATCTGTGCCACT	CCGTGATGTTCTGGAGCAGTGTTC	95	XM_003437924.4
*cox-2*	TGCTGAAAGAGGTCCACCCATACT	CGCTCAGATGCTGCACGTAGTC	99	XM_031758642.2
*β-Actin*	AGCCTTCCTTCCTTGGTATGGAAT	TGTTGGCGTACAGGTCCTTACG	94	KJ126772.1
*18s*	CCGGGTATGCGTGCATTTAT	CTGATTCCCCGTTACCCGTG	103	XR_003216134.1

*Note*: *bax*, bcl2-associated x protein; *18s*, 18s ribosomal RNA; *xbp1s*, spliced x-box binding protein 1.

Abbreviations: *β-actin*, beta-actin; *bcl2*, b-cell lymphoma 2; *caspase* 3, cysteine-aspartic protease 3; *caspase* 8, cysteine-aspartic protease 8; *caspase* 9, cysteine-aspartic protease 9; *chop*, c/ebp homologous protein; *cox-2*, cyclooxygenase-2; *crt*, calreticulin; *grp78*, glucose-regulated protein 78; *il-1β*, interleukin-1 beta; *il-12*, interleukin-12; *ire*, inositol-requiring enzyme; *mif*, macrophage migration inhibitory factor; *perk*, protein kinase r-like endoplasmic reticulum kinase; *tgf-β1*, transforming growth factor beta 1; *tnf-α*, tumor necrosis factor alpha.

## Data Availability

The datasets generated during the current study are available from the corresponding author upon reasonable request.
